# Relationships between serum angiogenesis markers and sarcopenia: a cross-sectional study

**DOI:** 10.3389/fmed.2026.1922045

**Published:** 2026-10-16

**Authors:** Hui Li, Huaqing Liu, Yanjuan Liu, Wentao Shi, Lingwei Shi, Feng Jia

**Affiliations:** 1 Department of Gastroenterology and General Surgery, Nanyang Central Hospital, Nanyang, Henan, China; 2 Department of Cardiology, Seventh People’s Hospital of Zhengzhou, Zhengzhou, Henan, China

**Keywords:** angiogenesis, ANGPT1, ANGPT2, sarcopenia, VEGF

## Abstract

**Background:**

This study investigates the associations between serum angiogenesis markers (VEGF, ANGPT1, and ANGPT2) and sarcopenia in older adults.

**Methods:**

This cross-sectional study included 471 older individuals admitted to Nanyang Central Hospital from May 2019 to May 2024. Sarcopenia was diagnosed using the Asian Working Group for Sarcopenia (AWGS) criteria, based on hand grip strength (HGS) and appendicular skeletal muscle mass (ASM). Serum levels of VEGF, ANGPT1, and ANGPT2 were measured, and statistical analyses included ROC curve analyses and logistic regression models to assess the relationships between angiogenesis markers and sarcopenia traits.

**Results:**

A total of 68 participants (14.44%) were diagnosed with sarcopenia. Significant decreases in VEGF and ANGPT1 levels were observed in the sarcopenia group compared to the normal cohort. Linear regression models showed that VEGF was positively correlated with HGS and ASM, while ANGPT1 was positively correlated with ASM. ROC curves indicated modest discriminatory ability for sarcopenia for both VEGF and ANGPT1. Logistic regression analysis revealed that low VEGF levels were associated with a higher risk of low HGS, low ASM, and sarcopenia, while low ANGPT1 levels were associated with increased risk of low ASM and sarcopenia (OR = 3.645, *p* < 0.001).

**Conclusions:**

VEGF and ANGPT1 are significantly associated with sarcopenia and may provide incremental value to standard clinical assessments. Further longitudinal studies are needed to elucidate the underlying mechanisms linking angiogenesis markers to sarcopenia.

## Introduction

Sarcopenia, characterized by the progressive loss of skeletal muscle mass and function, is a prevalent geriatric syndrome with significant impacts on individual mobility, quality of life, and overall health outcomes. As the global population continues to age, the prevalence of sarcopenia is expected to rise, necessitating a deeper understanding of the underlying mechanisms driving its development (1). While previous research has primarily focused on factors such as neuromuscular changes, protein metabolism, inflammation, physical inactivity, and lifestyle as contributors to sarcopenia, emerging evidence suggests that angiogenesis may play a critical yet underappreciated role in its development (2–4) Angiogenesis, the formation of new blood vessels from pre-existing ones, is essential for maintaining muscle health and function (5, 6). The microcirculation within muscles, determined largely by local angiogenic factors such as vascular endothelial growth factor (VEGF) and angiopoietin, ensures the delivery of oxygen and nutrients necessary for muscle metabolism and repair (7, 8). The decline in angiogenesis may lead to inadequate blood supply to muscles, contributing to muscle atrophy and weakness (9).

Furthermore, the role of angiogenesis in sarcopenia extends beyond mere blood supply. Angiogenic factors are involved in muscle regeneration and repair processes (10). However, with aging, the levels of these angiogenic factors diminish, potentially impairing the muscle's regenerative capacity (11, 12). Additionally, the interactions between angiogenesis and other biological processes, such as hormonal regulation and stem cell function, may also influence sarcopenia progression (13, 14). For example, androgens and estrogens have been found to upregulate VEGF expression, suggesting a link between hormonal decline and reduced angiogenesis in aging muscles (15).

Elucidating the mechanisms by which impaired angiogenesis contributes to sarcopenia is crucial for identifying novel therapeutic strategies. Although the significance of angiogenesis in muscle health is increasingly acknowledged, studies examining the associations between angiogenesis markers and sarcopenia at the population level remain limited. This study aims to address this gap by exploring the relationships between serum angiogenesis markers and sarcopenia, with a particular focus on the roles of angiogenesis in the development of this condition. Specifically, we examine three key serum angiogenesis markers—VEGF, angiopoietin-1 (ANGPT1), and angiopoietin-2 (ANGPT2)—in older individuals. VEGF plays a critical role in the formation of new blood vessels, whereas ANGPT1 and ANGPT2 are involved in regulating vessel stability and remodeling. By evaluating the associations between these systemic markers and sarcopenia, our study seeks to provide valuable insights into the clinical profile of this condition. These findings may ultimately contribute to a better understanding of the physiological changes associated with sarcopenia, thereby facilitating more comprehensive clinical assessments for the elderly population.

## Methods

### Study design

This study is a cross-sectional study conducted at the Department of Gastroenterology and General Surgery, Nanyang Central Hospital. The study was conducted in accordance with the tenets delineated in the Declaration of Helsinki and obtained approval from the Ethics Committee of Nanyang Central Hospital (ID: 20190771). We included all eligible elderly individuals admitted to our department between May 2019 and May 2024. The primary reasons for hospital admission mainly included common non-malignant gastrointestinal disorders, biliary tract diseases, and mild general surgical conditions. The inclusion criteria were: a. age ≥ 65 years; b. with written consent. The exclusion criteria were: a. with acute fractures; b. with malignant tumor; c. unavailable measurements of sarcopenia; d. unavailable measurement of angiogenesis markers. Participants fulfilling the specified inclusion and exclusion criteria were ultimately recruited for this investigation (Figure 1). Rigorous protocols were employed to ensure patient anonymity, and informed written consent was obtained from each participant prior to enrollment.

**Figure 1:**
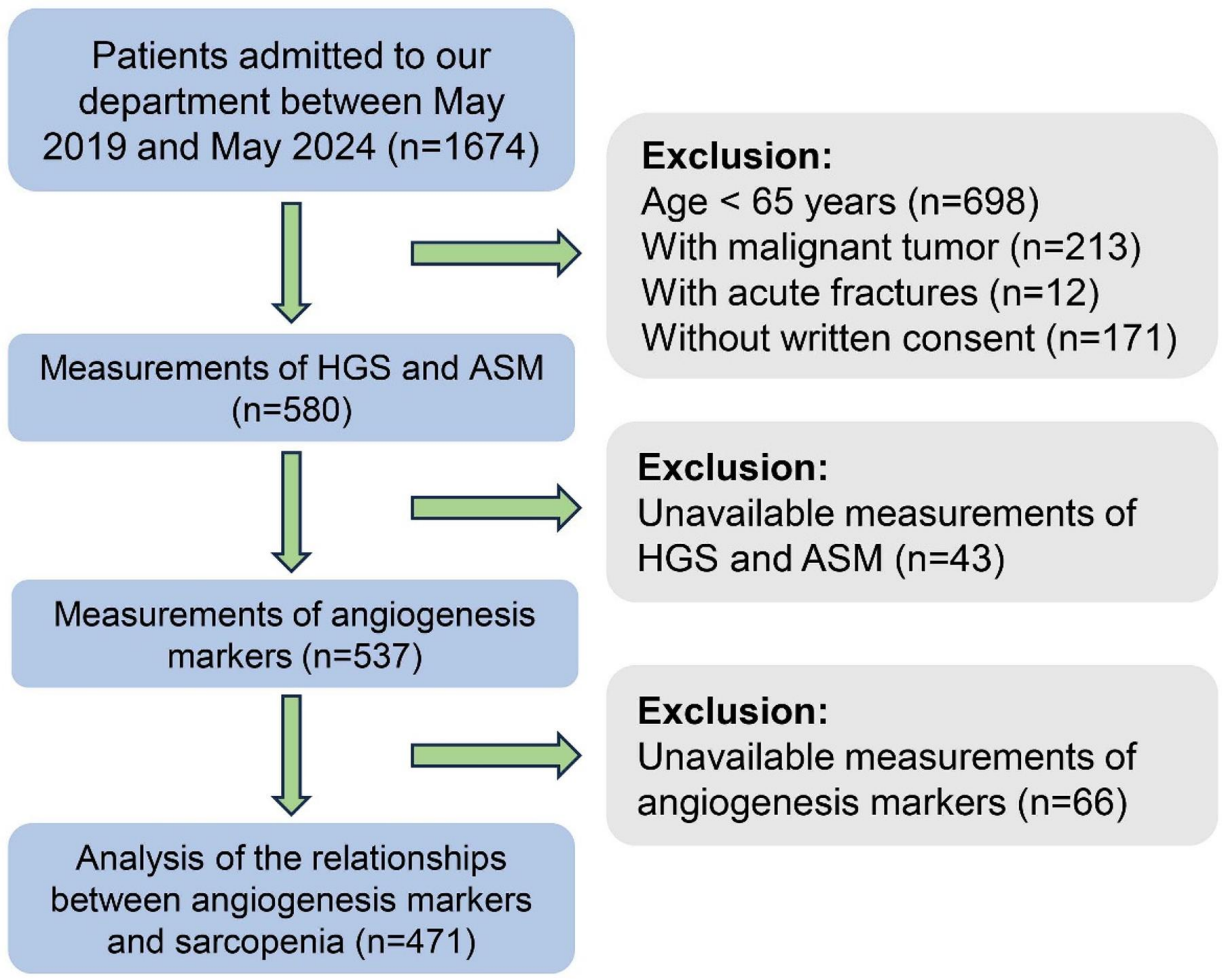
Flow chart of our study.

### Baseline data

Baseline characteristics of the older individuals were retrieved from the electronic medical records of our institution. These included demographic data (age, sex), anthropometric measures (height and weight), medical history (smoking and alcohol consumption habits, comorbid conditions), and clinical evaluations (electrocardiograms, chest x-rays). The Charlson Comorbidity Index (CCI) was utilized to assess the burden of comorbidities (16). Upon admission, laboratory assessments were performed to measure red blood cell count (RBC), hemoglobin (Hb), blood glucose (GLU), and albumin (ALB) levels.

### Angiogenesis markers

We examined and quantified three key serum angiogenesis markers—vascular endothelial growth factor (VEGF), angiopoietin-1 (ANGPT1), and angiopoietin-2 (ANGPT2)—in this cohort. Quantitative measurement of these markers was performed utilizing dedicated ELISA kits: a human VEGF ELISA kit (PV963, Beyotime), an ANGPT1 ELISA kit (JL10166-96 T, Jonlnbio), and an ANGPT2 ELISA kit (JL10504-96 T, Jonlnbio), strictly adhering to the manufacturers' protocols. Fasting venous blood samples were collected from participants on the morning following admission. Samples were allowed to clot at room temperature for 30 min before being centrifuged at 1,000 × g for 15 min at 4 °C. The separated serum was subsequently aliquoted and stored at −80 °C until analysis to ensure purity and eliminate any potential assay interferences. To prevent protein degradation, repeated freeze-thaw cycles were strictly avoided, and samples underwent only a single thaw immediately prior to analysis. Storage duration varied depending on the recruitment date, up to a maximum of 3 month. All ELISA assays were performed in duplicate to guarantee measurement reliability. Furthermore, the laboratory personnel conducting the assays were strictly blinded to the participants' clinical characteristics and sarcopenia status. The samples were applied to microplates pre-coated with capture antibodies specific to each marker. After incubation and thorough washing, the bound analytes were detected via HRP-conjugated secondary antibodies and a chromogenic substrate. The resultant absorbance was measured spectrophotometrically, and the quantitative concentrations of VEGF, ANGPT1, and ANGPT2 were determined by interpolation from a calibration curve constructed using serial dilutions of known standards.

Because data collection spanned five years, samples were analyzed in multiple batches. Throughout the entire study period, we exclusively utilized ELISA kits from the same manufacturers. Although the specific production dates and lot numbers varied over time, the manufacturers' standardized production processes and protocols remained strictly unchanged, ensuring the fundamental consistency of the assays. To strictly control for potential batch effects, we included standard internal quality control samples and reference calibration curves on every microplate. Measurement reliability was verified by calculating the coefficients of variation (CVs); the intra-assay CV was consistently <10%, and the inter-assay CV was <15%, ensuring robust reproducibility across all testing batches.

### Sarcopenia

The diagnosis of sarcopenia was determined in accordance with the guidelines set forth by the Asian Working Group for Sarcopenia (AWGS) (17). Sarcopenia was identified in patients who exhibited both reduced hand grip strength (HGS) and diminished appendicular skeletal muscle mass (ASM). Because standardized physical performance measurements were not routinely collected upon hospital admission due to patients' acute clinical conditions, we were able to confirm the diagnosis of sarcopenia but were unable to further categorize its severity. Specifically, HGS was classified as low if it was below 28 kg for men and below 18 kg for women. ASM was considered insufficient if it was less than 7.00 kg/m^2^ for men and less than 5.70 kg/m^2^ for women.

HGS was evaluated using a spring-loaded dynamometer that underwent routine weekly calibration. Measurements were taken with the patient in a seated position, with the shoulder adducted and neutrally rotated, the elbow flexed at 90 degrees, and the forearm and wrist in a neutral position. Both hands were tested. Each participant's HGS was assessed three times per hand with a one-minute rest period between trials to prevent muscle fatigue, and the absolute highest value recorded from either hand was documented for subsequent analysis.

To evaluate ASM, we employed bioimpedance analysis (BIA) using the InBody BWA2.0 device (InBody Co. Ltd., Shanghai, China). Recognizing that BIA measurements can be influenced by fluid status—particularly in hospitalized cohorts—strict measurement conditions were enforced. Assessments were conducted in the morning following an overnight fast and after the patients had emptied their bladders. Participants were required to rest for at least 15 min prior to the measurement and had refrained from any rigorous physical activity. Patients with clinically evident severe edema or overt hydration imbalances were excluded to ensure the accuracy of the ASM calculation. Specifically, multiple bioimpedance measurements were obtained across eight discrete frequencies—namely, 1 kHz, 5 kHz, 50 kHz, 250 kHz, 500 kHz, 1 MHz, 2 MHz, and 3 MHz—and were distributed across four major body segments: the right upper limb, left upper limb, right lower limb, and left lower limb. The device's proprietary algorithm subsequently processed these measurements to calculate ASM automatically. This calculated value was normalized by squaring the individual's height to account for variability in physical stature and ensure accurate comparisons among participants.

### Statistical analysis

In this study, continuous variables are presented as mean ± standard deviation, while categorical variables are shown as counts with corresponding percentages. For continuous variables, those conforming to a normal distribution were analyzed via independent Student's t-tests, whereas non-parametric data were evaluated using Wilcoxon rank-sum tests. Categorical variables were assessed using Chi-squared tests or Fisher's exact tests, depending on the data appropriateness.

Receiver operating characteristic (ROC) curves were constructed to evaluate the relationships between these markers and sarcopenia, with optimal cutoff values determined using the Youden index. Based on these cutoffs, patients were stratified into normal and elevated marker level groups, and sarcopenia traits were compared between these groups. To mitigate the risk of over-optimism and prevent data leakage, internal validation was performed using a strict 10-fold cross-validation approach. Specifically, within each iteration of the cross-validation process, the optimal cutoff value was dynamically determined via the Youden index using solely the training data within that specific fold. This fold-specific cutoff was subsequently applied to the independent validation fold to calculate the cross-validated sensitivity, specificity, and Area Under the Curve (AUC). Furthermore, to evaluate whether these markers provide incremental prognostic value over standard clinical characteristics, we established a base clinical model comprising age, sex, BMI, CCI, hemoglobin, albumin, smoking history, alcoholism history, and hypertension. We then compared this base model to full models incorporating the dichotomized biomarkers using the Likelihood Ratio Test (LRT) and the Akaike Information Criterion (AIC). Linear and logistic regression models were employed to further investigate the associations of VEGF, ANGPT1, and ANGPT2 with HGS, ASM, and sarcopenia. To rigorously control for potential confounding effects, we constructed two multivariate models. The criteria for selecting covariates in these models were based on their clinical relevance and their established or potential roles as confounding factors in the pathogenesis of sarcopenia. Model 1 was adjusted for fundamental demographic and anthropometric parameters, specifically age, sex, and BMI. As a sensitivity analysis, a comprehensive model (Model 2) was constructed with further adjustments for additional clinically relevant variables reflecting comorbidity burden, nutritional status, and lifestyle factors. These included the Charlson Comorbidity Index (CCI), hemoglobin (Hb), albumin (ALB), smoking history, alcoholism history, and hypertension. A *P* value below 0.05 was considered statistically significant. All analyses were performed using R software, version 4.1.2 (R Foundation for Statistical Computing, Vienna, Austria).

## Results

### Populations

Following the application of inclusion and exclusion criteria and the measurement of angiogenesis markers and sarcopenia characteristics, this study ultimately included 471 patients (Figure 1), of whom 68 were diagnosed with sarcopenia. In the non-sarcopenia group, the mean age was 72.84 ± 6.28 years, the mean BMI was 20.62 ± 4.93 kg/m^2^, and 208 patients (51.61%) were female. In the sarcopenia group, the mean age was 72.79 ± 5.94 years, the mean BMI was 20.64 ± 4.91 kg/m^2^, and 30 patients (44.12%) were female. Baseline characteristics showed no significant differences between the two groups (all *p* > 0.05, Table 1).

**Table 1:** Baseline features of the individuals included in this study.

Variables	Overall	Normal	Sarcopenia	*p* value
	(*n* = 471)	(*n* = 403)	(*n* = 68)	
Age(years)	72.83 ± 6.23	72.84 ± 6.28	72.79 ± 5.94	0.942
BMI (kg/m2)	20.62 ± 4.92	20.62 ± 4.93	20.64 ± 4.91	0.643
Sex (female)	238 (50.53%)	208 (51.61%)	30 (44.12%)	0.253
Smoking history (yes)	114 (24.20%)	99 (24.57%)	15 (22.06%)	0.655
Alcoholism history(yes)	83 (17.62%)	71 (17.62%)	12 (17.65%)	0.995
CCI score (>4)	164 (34.82%)	139 (34.49%)	25 (36.76%)	0.716
Electrocardiogram (abnormal)	126 (26.75%)	106 (26.30%)	20 (29.41%)	0.592
Chest radiograph (abnormal)	113 (23.99%)	97 (24.07%)	16 (23.53%)	0.923
Hypertension(yes)	174 (36.94%)	153 (37.97%)	21 (30.88%)	0.263
RBC (10^12/L)	4.22 ± 1.16	4.22 ± 1.16	4.18 ± 1.16	0.515
Hb (g/L)	91.20 ± 28.20	91.63 ± 28.44	88.65 ± 26.83	0.393
ALB (g/L)S	36.23 ± 11.76	36.17 ± 11.65	36.55 ± 12.49	0.976
GLU (mmol/L)	5.33 ± 1.57	5.32 ± 1.54	5.41 ± 1.74	0.828
VEGF (pg/mL)	135.47 ± 60.72	138.60 ± 60.89	116.95 ± 56.64	<0.001
ANGPT1 (ng/mL)	32.64 ± 19.25	34.15 ± 19.33	23.68 ± 16.15	<0.001
ANGPT2 (ng/mL)	3.09 ± 1.50	3.10 ± 1.51	3.06 ± 1.43	0.884

BMI, Body Mass Index; CCI, Charlson Comorbidity Index; RBC, Red Blood Cell; GLU, Blood Glucose; Hb, Hemoglobin; ALB, Albumin; VEGF, Vascular Endothelial Growth Factor; ANGPT1, Angiopoietin 1; ANGPT2, Angiopoietin 2.

### Angiogenesis markers of individuals with different sarcopenia traits

Patients meeting the criteria for low HGS, low ASM, and sarcopenia were identified based on AWGS guidelines. Subsequent comparisons of angiogenesis marker concentrations between these populations and the normal cohort yielded the following results (Figure 2): The low HGS group demonstrated a significant decrease in VEGF levels relative to the normal group (*p* = 0.006), whereas ANGPT1 and ANGPT2 concentrations did not differ significantly. The low ASM group exhibited lower VEGF levels compared to the general group (*p* < 0.001), with ANGPT1 and ANGPT2 levels remaining equivalent. Interestingly, in the sarcopenia group, both VEGF and ANGPT1 levels were notably diminished compared to the normal group, suggesting a more substantial decline in these markers among sarcopenic patients.

**Figure 2:**
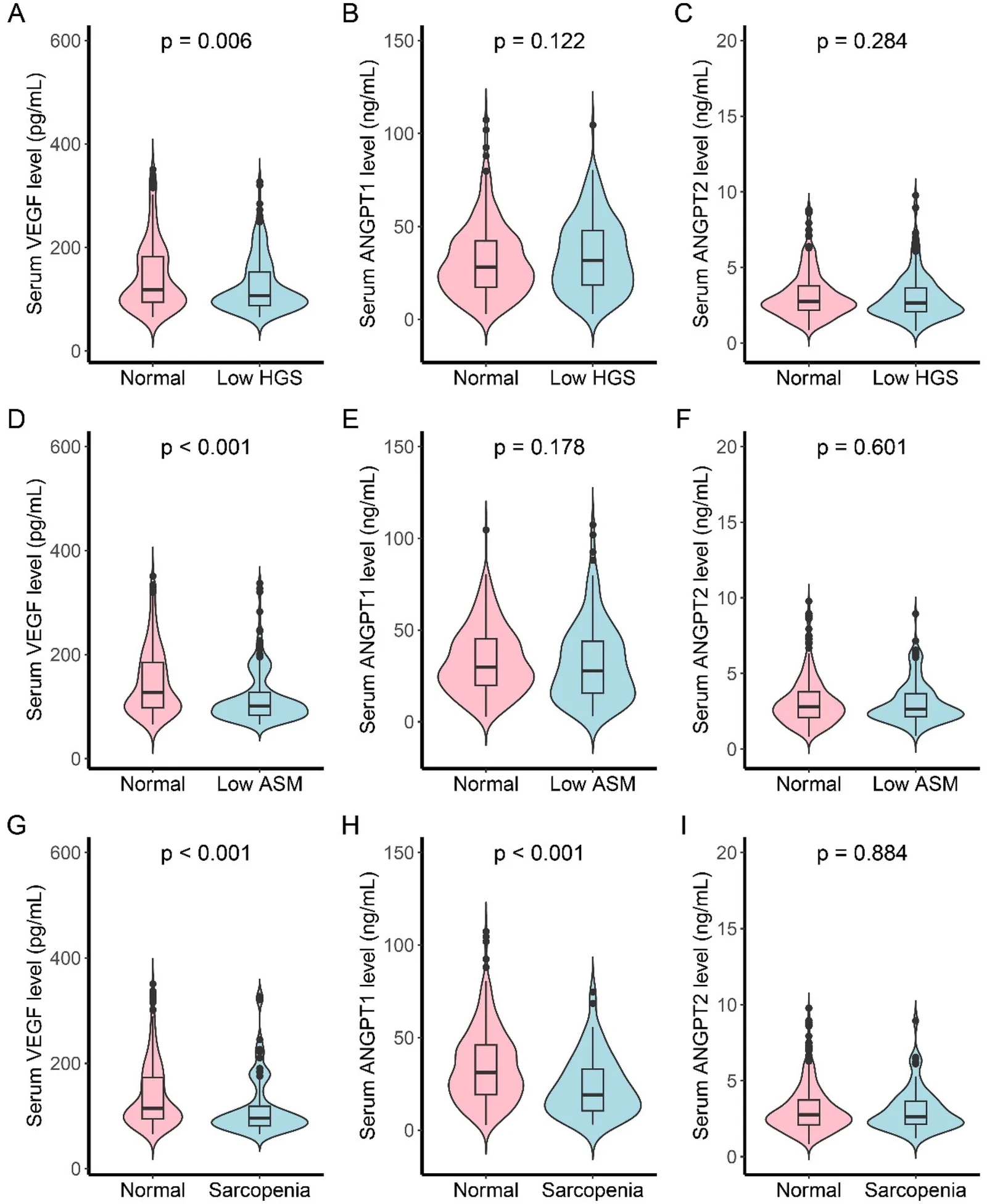
VEGF, ANGPT1, and ANGPT2 levels of individuals with different sarcopenia traits. **A-C:** Comparison of VEGF, ANGPT1, and ANGPT2 levels between individuals with normal HGS (*n* = 255) and low HGS (*n* = 216); **D-F:** Comparison of VEGF, ANGPT1, and ANGPT2 levels between individuals with normal ASM (*n* = 288) and low ASM (*n* = 183); **G-I:** Comparison of VEGF, ANGPT1, and ANGPT2 levels between normal individuals (*n* = 403) and individuals with sarcopenia (*n* = 68). Due to the non-normal distribution of serum biomarker data, all *p*-values were calculated using the non-parametric Wilcoxon rank-sum test.

To further explore the relationships between these markers and HGS/ASM, linear regression models were constructed. Initially, univariate models were created with the markers as variables. Subsequently, Multivariate Model 1 was adjusted for age, sex, and BMI, while Model 2 was additionally adjusted for CCI, Hb, ALB, smoking history, alcoholism history, and hypertension as a sensitivity analysis. As shown in Table 2, VEGF was positively correlated with HGS and ASM in the univariate analysis (*p* < 0.001 for both), and these significant associations consistently persisted in both Model 1 and the fully adjusted Model 2 (for Model 2 HGS: β  = 3.186, *p* < 0.001; ASM: β  = 1.358, *p* < 0.001). For ANGPT1, while the univariate model and Model 1 only showed a significant positive correlation with ASM (*p* < 0.01 for both), the fully adjusted Model 2 demonstrated significant positive correlations with both ASM (β = 0.488, *p* < 0.001) and HGS (β = 1.275, *p* = 0.044). ANGPT2 showed no significant correlations with sarcopenia traits across any of the models.

**Table 2:** Univariate and multivariate linear models of VEGF, ANGPT1, and ANGPT2 for HGS and ASM.

Variables	HGS					
	Univariate model		Multivariate model 1		Multivariate model 2	
	*β* [95%CI]	p	β [95%CI]	p	β [95%CI]	p
VEGF (per 1 SD increase)	3.095 [1.950, 4.240]	<0.001	3.241 [2.090, 4.392]	<0.001	3.186 [2.031, 4.341]	<0.001
ANGPT1 (per 1 SD increase)	0.799 [−0.377, 1.976]	0.183	1.161 [−0.073, 2.394]	0.065	1.275 [0.037, 2.514]	0.044
ANGPT2 (per 1 SD increase)	0.691 [−0.486, 1.868]	0.249	0.601 [−0.593, 1.795]	0.323	0.505 [−0.701, 1.711]	0.411

Variables	ASM					
	Univariate model		Multivariate model 1		Multivariate model 2	
	β [95%CI]	p	β [95%CI]	p	β [95%CI]	p
VEGF (per 1 SD increase)	1.340 [1.118, 1.562]	<0.001	1.360 [1.140, 1.581]	<0.001	1.358 [1.137, 1.579]	<0.001
ANGPT1 (per 1 SD increase)	0.413 [0.163, 0.663]	0.001	0.487 [0.227, 0.746]	<0.001	0.488 [0.227, 0.749]	<0.001
ANGPT2 (per 1 SD increase)	0.125 [−0.127, 0.378]	0.33	0.084 [−0.170, 0.337]	0.518	0.105 [−0.151, 0.361]	0.422

HGS, Hand Grip Strength; ASM, Appendicular Skeletal Muscle Mass; VEGF, Vascular Endothelial Growth Factor; ANGPT1, Angiopoietin 1; ANGPT2, Angiopoietin 2. β represents the unstandardized linear regression coefficient corresponding to a 1 Standard Deviation (SD) increase in the continuous biomarker.

### Diagnostic values of angiogenesis markers in sarcopenia traits

For a comprehensive diagnostic assessment of the markers, ROC curve analyses were conducted for conditions including low HGS, low ASM, and sarcopenia. Figure 3 presents the ROC curves for sarcopenia prediction using VEGF, ANGPT1, and ANGPT2. Both VEGF and ANGPT1 showed modest predictive values for sarcopenia (AUROCs of 0.641 for VEGF, 0.668 for ANGPT1). Employing the Youden index, optimal cutoff thresholds for identifying sarcopenia were established from the ROC curves for each marker: VEGF ≤ 102.55 pg/mL, ANGPT1 ≤ 19.47 ng/mL, and ANGPT2 ≤ 2.37 ng/mL. Consequently, patients were stratified into subgroups characterized by low concentrations of these markers. These cutoffs, along with their corresponding sensitivity and specificity values, are summarized in Supplementary Table 1.

**Figure 3:**
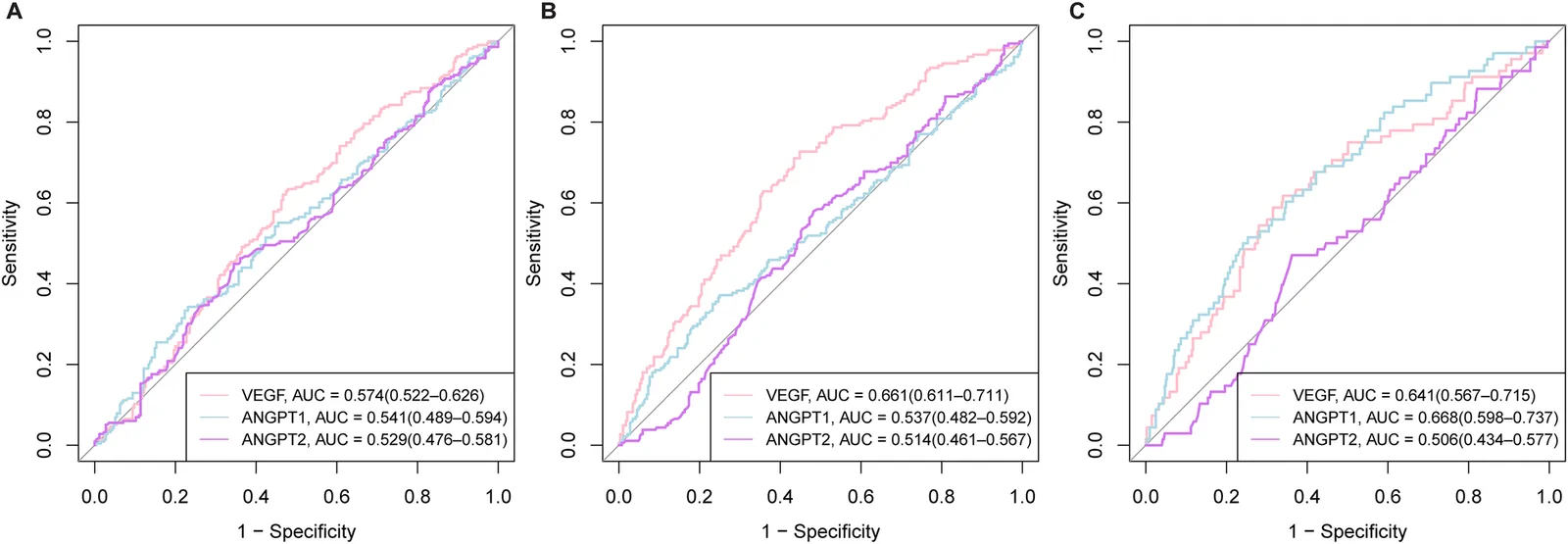
ROC curves of VEGF, ANGPT1, and ANGPT2 levels for low HGS, low ASM, and sarcopenia. **A:** for HGS; **B:** for ASM; **C:** sarcopenia.

To internally validate these diagnostic cut-offs and address potential overestimation, a 10-fold cross-validation was performed (Supplementary Table 2). The cross-validated AUCs remained highly consistent (0.643 for VEGF and 0.654 for ANGPT1), with stable cross-validated sensitivity and specificity, confirming the internal validity of these markers. Furthermore, we assessed whether these markers add useful information to standard clinical evaluations. Compared to a base clinical model, incorporating either low VEGF or low ANGPT1 status significantly improved the model's goodness-of-fit (Supplementary Table 3), as evidenced by significant Likelihood Ratio Test results (*p* = 0.002 for VEGF; *p* < 0.001 for ANGPT1) and corresponding reductions in AIC values (*Δ*AIC = 7.99 and 23.32, respectively). ANGPT2 did not provide any significant incremental value (*p* = 0.986).

### Relationships between angiogenesis markers and sarcopenia

Based on the cut-off values of the three angiogenic markers mentioned above, we compared the sarcopenia characteristics among different groups of individuals (Table 3). For VEGF, the low VEGF group showed significantly lower HGS, ASM, and a higher risk of low HGS, low ASM, and sarcopenia compared to the control group. The results for ANGPT1 were similar, with significant differences observed in HGS, ASM, and the incidence of sarcopenia. In contrast, stratification based on ANGPT2 was only associated with low HGS.

**Table 3:** Sarcopenia traits of individuals grouped by VEGF, ANGPT1, and ANGPT2.

Sarcopenia traits	Overall	Normal VEGF	Low VEGF	p	Normal ANGPT1	Low ANGPT1	p	Normal ANGPT2	Low ANGPT2	p
	(*n* = 471)	(*n* = 292)	(*n* = 179)		(*n* = 334)	(*n* = 137)		(*n* = 293)	(*n* = 178)	
HGS (kg)	25.92 ± 12.99	27.42 ± 13.25	23.46 ± 12.21	0.001	26.82 ± 13.31	23.70 ± 11.94	0.018	26.45 ± 13.43	25.04 ± 12.21	0.255
ASM (kg/m2)	7.36 ± 2.79	8.08 ± 2.64	6.18 ± 2.62	<0.001	7.80 ± 2.77	6.27 ± 2.52	<0.001	7.45 ± 2.91	7.20 ± 2.58	0.331
Low HGS	216 (45.86%)	121 (41.44%)	95 (53.07%)	0.014	155 (46.41%)	61 (44.53%)	0.71	124 (42.32%)	92 (51.69%)	0.048
Low ASM	183 (38.85%)	90 (30.82%)	93 (51.96%)	<0.001	117 (35.03%)	66 (48.18%)	0.008	107 (36.52%)	76 (42.70%)	0.182
Sarcopenia	68 (14.44%)	26 (8.90%)	42 (23.46%)	<0.001	33 (9.88%)	35 (25.55%)	<0.001	36 (12.29%)	32 (17.98%)	0.088

HGS, Hand Grip Strength; ASM, Appendicular Skeletal Muscle Mass; VEGF, Vascular Endothelial Growth Factor; ANGPT1, Angiopoietin 1; ANGPT2, Angiopoietin 2.

To control for potential confounding effects, we performed univariate and two multivariate logistic regression analyses (Table 4). Model 1 was adjusted for age, gender, and BMI. To confirm the robustness of our findings, a sensitivity analysis (Model 2) was conducted with further adjustments for CCI, Hb, ALB, smoking history, alcoholism history, and hypertension. In the univariate analysis, low VEGF was significantly associated with low HGS, low ASM, and sarcopenia. These associations remained robust across both Model 1 and the fully adjusted Model 2 (for low VEGF and sarcopenia in Model 2: OR = 3.360, 95% CI: 1.968–5.844, *p* < 0.001). Similarly, low ANGPT1 was significantly associated with an increased risk of low ASM and sarcopenia in the univariate analysis, Model 1, and Model 2 (for low ANGPT1 and sarcopenia in Model 2: OR = 3.709, 95% CI: 2.125–6.539, *p* < 0.001). Although low ANGPT2 showed a marginal association with low HGS in the univariate model (OR = 1.458, *p* = 0.048), this significance was completely lost after adjusting for covariates in Model 1 and Model 2.

**Table 4:** Univariate and multivariate logistic models of VEGF, ANGPT1, and ANGPT2 for sarcopenia traits.

Variables	Univariate model					
	Low HGS		Low ASM		Sarcopenia	
	OR [95%CI]	p	OR [95%CI]	p	OR [95%CI]	p
VEGF (per 1 SD increase)	0.733 [0.601, 0.886]	0.002	0.547 [0.429, 0.683]	<0.001	0.638 [0.448, 0.870]	0.008
ANGPT1 (per 1 SD increase)	1.141 [0.952, 1.371]	0.155	0.940 [0.778, 1.131]	0.513	0.505 [0.358, 0.693]	<0.001
ANGPT2 (per 1 SD increase)	0.921 [0.765, 1.105]	0.379	0.936 [0.773, 1.127]	0.488	0.977 [0.743, 1.254]	0.859
Low VEGF	1.598 [1.100, 2.328]	0.014	2.427 [1.655, 3.573]	<0.001	3.136 [1.857, 5.388]	<0.001
Low ANGPT1	0.927 [0.620, 1.381]	0.71	1.724 [1.151, 2.583]	0.008	3.130 [1.850, 5.314]	<0.001
Low ANGPT2	1.458 [1.003, 2.122]	0.048	1.295 [0.885, 1.895]	0.183	1.565 [0.929, 2.626]	0.09

Variables	Multivariate model 1					
	Low HGS		Low ASM		Sarcopenia	
	OR [95%CI]	p	OR [95%CI]	p	OR [95%CI]	p
VEGF (per 1 SD increase)	0.670 [0.542, 0.820]	<0.001	0.501 [0.388, 0.636]	<0.001	0.617 [0.431, 0.847]	0.005
ANGPT1 (per 1 SD increase)	0.991 [0.814, 1.205]	0.932	0.843 [0.685, 1.030]	0.098	0.439 [0.304, 0.615]	<0.001
ANGPT2 (per 1 SD increase)	0.984 [0.813, 1.188]	0.866	0.992 [0.816, 1.202]	0.938	0.999 [0.759, 1.285]	0.995
Low VEGF	1.824 [1.235, 2.706]	0.003	2.734 [1.836, 4.100]	<0.001	3.292 [1.939, 5.691]	<0.001
Low ANGPT1	1.148 [0.754, 1.752]	0.52	2.199 [1.432, 3.400]	<0.001	3.645 [2.105, 6.364]	<0.001
Low ANGPT2	1.177 [0.793, 1.746]	0.417	1.099 [0.736, 1.637]	0.641	1.489 [0.869, 2.543]	0.145

Variables	Multivariate model 2					
	Low HGS		Low ASM		Sarcopenia	
	OR [95%CI]	p	OR [95%CI]	p	OR [95%CI]	p
VEGF (per 1 SD increase)	0.671 [0.542, 0.823]	<0.001	0.489 [0.376, 0.623]	<0.001	0.609 [0.424, 0.837]	0.004
ANGPT1 (per 1 SD increase)	0.972 [0.796, 1.186]	0.783	0.853 [0.692, 1.047]	0.133	0.436 [0.301, 0.615]	<0.001
ANGPT2 (per 1 SD increase)	0.999 [0.822, 1.212]	0.99	0.961 [0.787, 1.169]	0.695	0.998 [0.757, 1.288]	0.986
Low VEGF	1.827 [1.230, 2.729]	0.003	2.816 [1.874, 4.267]	<0.001	3.360 [1.968, 5.844]	<0.001
Low ANGPT1	1.229 [0.801, 1.889]	0.345	2.179 [1.404, 3.404]	0.001	3.709 [2.125, 6.539]	<0.001
Low ANGPT2	1.154 [0.772, 1.721]	0.483	1.124 [0.746, 1.688]	0.574	1.479 [0.861, 2.535]	0.154

HGS, Hand Grip Strength; ASM, Appendicular Skeletal Muscle Mass; VEGF. Vascular Endothelial Growth Factor; ANGPT1, Angiopoietin 1; ANGPT2, Angiopoietin 2. For continuous variables, Odds Ratios (OR) represent the risk associated with a 1 Standard Deviation (SD) increase in the respective biomarker.

## Discussion

The purpose of this study was to explore the relationships between serum angiogenesis markers and sarcopenia in older individuals. Our findings revealed significant associations between VEGF and ANGPT1 levels and sarcopenia traits. Specifically, lower VEGF levels were consistently linked to low HGS, low ASM, and sarcopenia, whereas lower ANGPT1 levels were primarily associated with low ASM and an increased risk of overall sarcopenia. These results highlight the potential roles of angiogenesis in the pathogenesis of sarcopenia and suggest these markers are robustly associated with sarcopenia and may improve the statistical fit of existing clinical risk models.

Angiogenesis plays a crucial role in skeletal muscle regeneration. In the elderly population, it is hypothesized that sarcopenia, which is the age-related loss of skeletal muscle mass and function, often occurs because chronic injuries to skeletal muscles fail to heal properly (18). Angiogenesis is key to this healing process (19). Following skeletal muscle injury, the initial response involves tissue destruction and inflammation, characterized by infiltration of immune cells such as macrophages and neutrophils (6). These cells release pro-inflammatory cytokines and growth factors that initiate the healing process (20, 21). Angiogenesis is an early and essential event in muscle regeneration, preceding myogenesis (20, 22). The formation of new blood vessels from pre-existing ones provides a structural and functional framework for the delivery of nutrients and oxygen to the site of injury (23, 24). Therefore, the capacity for angiogenesis can serve as a potential indicator of the onset and progression of sarcopenia, as it reflects the body's ability to repair damaged muscle tissue through the formation of new blood vessels that support nutrient delivery and tissue maintenance.

VEGF is a critical factor in angiogenesis, promoting the formation of new blood vessels and ensuring adequate oxygen and nutrient supply to muscles (25). In our study, lower VEGF levels were consistently linked to reduced HGS and ASM, as well as a higher risk of sarcopenia. This aligns with previous research indicating that reduced capillary density and angiogenic factors in skeletal muscles contribute to muscle atrophy and weakness. Some studies have reported that delivering the VEGF gene to mice can effectively improve skeletal muscle ischemia and nerve regeneration, thereby promoting muscle injury repair (26). VEGF has also been shown to enhance skeletal muscle regeneration in rabbits (27). Similarly, VEGF may play a positive feedback role in skeletal muscle. Research indicates that exercise can lead to increased expression of VEGF in skeletal muscle, which may be associated with the physiological damage to skeletal muscle caused by exercise (11, 28). The increase in VEGF expression may further result in enhanced skeletal muscle function and overall physiological benefits (13). Similarly, studies have confirmed that massage can also increase the expression of VEGF in skeletal muscle (11, 29). Additionally, other research has demonstrated that exercise-induced changes in skeletal muscle VEGF can modulate the downstream FOXO signaling pathway, thereby promoting muscle hypertrophy (30). Similarly, VEGF can directly promote the regeneration of skeletal muscle stem cells, thereby facilitating muscle repair (31).

ANGPT1, which is involved in regulating vessel stability and remodeling, also demonstrated significant associations with sarcopenia traits. Lower ANGPT1 levels were strongly associated with low ASM and an increased risk of sarcopenia, though they did not independently predict low HGS in logistic regression models. ANGPT1 can significantly enhance skeletal muscle regeneration in mice, an effect that may be mediated through the induction of cell cycle regulation and myogenic programs in muscle progenitor cells (32). Similarly, ANGPT1 in skeletal muscle is regulated by exercise, a phenomenon that has been confirmed in both rats and humans (33, 34). A study in goats also suggests that ANGPT1 may promote muscle atrophy by reducing the differentiation of satellite cells in goat skeletal muscle (35).

Crucially, the link between microcirculation and muscle health is strongly supported by human clinical studies (36). Research utilizing human skeletal muscle biopsies has demonstrated that aging is associated with significant declines in capillary density, specifically reductions in the capillary-to-fiber ratio and capillary contacts per fiber (37). This capillary rarefaction is most pronounced around type II muscle fibers, which are selectively atrophied in sarcopenia (38). Furthermore, reduced capillarization in older adults has been clinically correlated with diminished appendicular muscle mass, lowered grip strength, and decreased physical performance (39, 40). While our study measures systemic markers rather than direct muscle capillarization, our findings align with the broader human evidence indicating that vascular health is intimately tied to the preservation of muscle mass and function during aging.

Building upon this structural decline, the reduction of circulating VEGF and ANGPT1 observed in sarcopenic individuals is mechanistically rooted in this progressive age-related capillary rarefaction. As the microvascular network deteriorates, the local endothelial cells diminish. This compromised vascular niche directly restricts satellite cell (SC) function, as endothelial cells and SCs are anatomically juxtaposed and rely heavily on reciprocal paracrine crosstalk (41). Specifically, VEGF acts directly on SC receptors to stimulate their initial activation, proliferation, and migration in response to muscle stress (31, 42). Following this activation phase, ANGPT1 is critical for orchestrating the later stages of the myogenic program; it not only supports SC survival but actively coordinates their differentiation and ultimate fusion into multinucleated mature myotubes (32, 35). Consequently, the simultaneous decline of VEGF and ANGPT1 in aging muscles dual-impairs the microenvironment: it restricts capillary blood supply and fundamentally disrupts the critical sequence of SC activation and fusion, thereby driving the regenerative failure that underpins the biology of sarcopenia.

In contrast, ANGPT2 did not show significant associations with sarcopenia characteristics in our study. ANGPT2 typically antagonizes ANGPT1 and is involved in vessel remodeling and destabilization. However, its role in sarcopenia remains unclear and may require further investigation, possibly in the context of dynamic changes in the ANGPT1/ANGPT2 ratio.

Our study has several strengths. First, it is one of the few hospital-based studies examining the relationships between serum angiogenesis markers and sarcopenia. By using standardized diagnostic criteria for sarcopenia and rigorous measurement protocols for angiogenesis markers, we were able to provide robust evidence of their associations. Second, our comprehensive statistical analyses, including ROC curve analyses and multivariate regression models, allowed us to control for potential confounding factors and assess the diagnostic value of these markers.

Nevertheless, some limitations should be acknowledged. The cross-sectional design of our study precludes establishing causality or determining the temporal sequence between changes in angiogenesis markers and the development of sarcopenia. Reverse causation remains a distinct possibility. Longitudinal studies are needed to further investigate these relationships. Furthermore, it is critical to emphasize that serum VEGF and ANGPT1 are systemic markers, not direct measurements of local angiogenesis within the skeletal muscle microenvironment. Their systemic concentrations may reflect a variety of confounding systemic factors, including subclinical underlying diseases, systemic inflammation, nutritional status, and overall physical activity levels. Additionally, our study focused on a specific population of older individuals admitted to a single hospital over a five-year period, rather than community-dwelling older adults. This single-center, hospital-based design introduces a potential selection bias and limits the generalizability of our findings to the broader, healthy community-dwelling elderly population. Future multicenter, longitudinal studies involving diverse, community-based cohorts are necessary to validate our results. Furthermore, our study lacked data on physical performance metrics. The absence of physical performance data precluded us from evaluating the severity of sarcopenia in our cohort. Moreover, Due to multiple comparisons, borderline *p*-values should be interpreted cautiously to avoid Type I errors, and these exploratory findings require independent validation. Finally, while we measured three key angiogenesis markers, the complex angiogenic process involves numerous other factors and interactions that were not accounted for in this study.

In conclusion, our findings emphasize the important roles of VEGF and ANGPT1 in sarcopenia and suggest their potential as associated clinical markers reflecting skeletal muscle health. While their independent diagnostic discrimination is modest, they significantly improve the statistical goodness-of-fit when incorporated into models based on standard clinical parameters. Further research is needed to explore the underlying mechanisms and to determine the clinical utility of these markers in the management of sarcopenia.

## Data Availability

The raw data supporting the conclusions of this article will be made available by the authors, without undue reservation.
